# Traffic-Related Air Pollution and Perinatal Mortality: A Case–Control Study

**DOI:** 10.1289/ehp.11679

**Published:** 2008-09-22

**Authors:** Andréa Paula Peneluppi de Medeiros, Nelson Gouveia, Reinaldo Paul Pérez Machado, Miriam Regina de Souza, Gizelton Pereira Alencar, Hillegonda Maria Dutilh Novaes, Márcia Furquim de Almeida

**Affiliations:** 1 Department of Preventive Medicine, Faculty of Medicine; 2 Department of Geography and; 3 Department of Epidemiology, Faculty of Public Health, University São Paulo, São Paulo, Brazil

**Keywords:** air pollution, epidemiology, geographic information systems, perinatal mortality, traffic density

## Abstract

**Background:**

Ambient levels of air pollution may affect the health of children, as indicated by studies of infant and perinatal mortality. Scientific evidence has also correlated low birth weight and preterm birth, which are important determinants of perinatal death, with air pollution. However, most of these studies used ambient concentrations measured at monitoring sites, which may not consider differential exposure to pollutants found at elevated concentrations near heavy-traffic roadways.

**Objectives:**

Our goal was to examine the association between traffic-related pollution and perinatal mortality.

**Methods:**

We used the information collected for a case–control study conducted in 14 districts in the City of São Paulo, Brazil, regarding risk factors for perinatal deaths. We geocoded the residential addresses of cases (fetal and early neonatal deaths) and controls (children who survived the 28th day of life) and calculated a distance-weighted traffic density (DWTD) measure considering all roads contained in a buffer surrounding these homes.

**Results:**

Logistic regression revealed a gradient of increasing risk of early neonatal death with higher exposure to traffic-related air pollution. Mothers exposed to the highest quartile of the DWTD compared with those less exposed exhibited approximately 50% increased risk (adjusted odds ratio = 1.47; 95% confidence interval, 0.67–3.19). Associations for fetal mortality were less consistent.

**Conclusions:**

These results suggest that motor vehicle exhaust exposures may be a risk factor for perinatal mortality.

Child mortality has decreased substantially in Brazil in past decades; nonetheless, it still has higher values than those found in developed countries (Vermelho et al. 2002). Perinatal deaths—those that occur *in utero* (≥22 weeks of gestation or fetal deaths) and in the first 6 days of life (early neonatal deaths)—contribute most to this situation. Perinatal mortality is an indicator of mother and child health and may reflect the conditions of reproductive health, which is related to socioeconomic position, quality of antenatal care, and delivery characteristics (Jackson et al. 1999). Some of the several risk factors identified for perinatal mortality include mother-related factors and complications in pregnancy, labor, and delivery, which may affect both the newborn and the fetus (e.g., maternal hypertension, placenta previa, premature placental detachment, and other morphologic and functional abnormalities of the placenta); respiratory and cardiovascular disorders specific to the perinatal period; infections; and disorders related to the length of pregnancy and fetal growth [e.g., intrauterine growth restriction (IUGR), preterm birth, and low birth weight] (Almeida et al. 2007; Conde-Agudelo et al. 2000; Kramer et al. 2002; Schoeps et al. 2007). More recently, studies have indicated that exposure to air pollution may be associated with low birth weight and preterm birth (Ritz and Yu 1999; Ritz et al. 2000), both of which are important risk factors for perinatal death. In addition, urban air pollution has also been directly implicated in perinatal mortality (Lin et al. 2004; Nishioka et al. 2000; Pereira et al. 1998). The association between deaths in the neonatal period (0–28 days of life) and air pollution was also identified in the studies by Lipfert et al. (2000) and Hajat et al. (2007).

However, these studies assessed the exposure to air pollution based on the average concentration of pollutants obtained from air quality monitoring stations. This direct measuring system reflects the levels of pollutants in a relatively large area. Although this procedure may adequately reflect average exposure of pregnant women to background levels of air pollution in their neighborhood, it does not take into account differences in exposure within neighborhoods because of proximity to heavy-traffic roadways and freeways. It is likely that those living closer to these sources may experience greater exposure to toxic compounds released directly in vehicle exhaust or formed in the atmosphere adjacent to roadways (Wilhelm and Ritz 2003). In addition, measurement studies have shown that concentrations of traffic-related air pollutants are elevated near roadways (Kinney et al. 2000; Lena et al. 2002).

Many studies have investigated the effects of air pollution on health using traffic-related exposure (Langholz et al. 2002; Pearson et al. 2000; Wilhelm and Ritz 2003) and found higher health effects among those living closer to streets with higher traffic volumes. Therefore, in the present study we aimed to investigate the association between perinatal mortality and maternal exposure to traffic-related air pollutants, assessed indirectly by calculating the distance-weighted traffic density (DWTD) in the vicinities of mothers’ homes, which we based on the subjects’ residential address, the distance of their homes to surrounding streets, and the traffic flow in these streets.

## Materials and Methods

We used information collected from a case–control study conducted in 14 districts located in the south region of São Paulo, Brazil, regarding risk factors for early fetal and neonatal deaths (Almeida et al. 2007; Schoeps et al. 2007). These districts account for approximately 23% of the population and 44% of the city’s geographic area (Almeida et al. 2004). This study was approved by the ethical committees of all institutions involved, and all participants gave informed consent before interviews.

### Subjects

The population studied comprised all births and fetal deaths from women living in this area in the period between 1 August 2000 and 31 January 2001. Cases were all perinatal deaths that occurred in the study period in the area identified through a linkage of the Brazilian Live Birth Information System (SINASC) and the Brazilian Mortality Information System (SIM) databases (both available at www.datasus.gov.br). Both systems have very high population coverage for São Paulo (Almeida et al. 2006). Controls were a random sample of all children who survived the 28th day of life, and were obtained through the same databases. We excluded all nonhospital deliveries. The initial sample was defined for a study on perinatal mortality; however, preliminary analysis of results indicated differences between risk factors for early neonatal and fetal deaths. Thus, we decided to analyze these components separately, maintaining the number of controls previously established.

Primary data were collected by home interviews up to 6 months after the event using a form containing variables regarding families’ socioeconomic characteristics, maternal reproductive history and risk factors, outcome of previous and present pregnancies and deliveries, and use of and access to health care. A protocol for collecting data from patient charts in the hospitals where deliveries took place was used for both cases and controls. For deaths that occurred in a different hospital (other than that of the delivery), data were also obtained from patient charts of those services.

### Exposure assessment

We geocoded the residential addresses of case and control mothers based on their postal code and street number using MapInfo (professional version 8.5; MapInfo Corporation, New York, NY, USA).

A digital cartographic base of the studied area was provided by the Traffic Engineering Company (CET) of the Department of Transport of the city of São Paulo. This contained a simulation of traffic flows on every segment of all freeways, arterials, and collectors attained from systematic counts conducted in the peak morning hours, from 0730 to 0830 hours. This data set also contained an estimate of the total traffic flow from all local roads inside predefined microareas (polygons). Therefore, for local roads without information on flows, we followed a procedure developed by CET that divides the estimated total flow for each polygon proportionally for each local road according to its length inside the area. We performed this procedure using Maptitude software (version 4.6; Calliper Corp., Newton, MA, USA), which enabled us to distribute and attribute traffic flow values for all local roads without counts.

**Figure 1:**
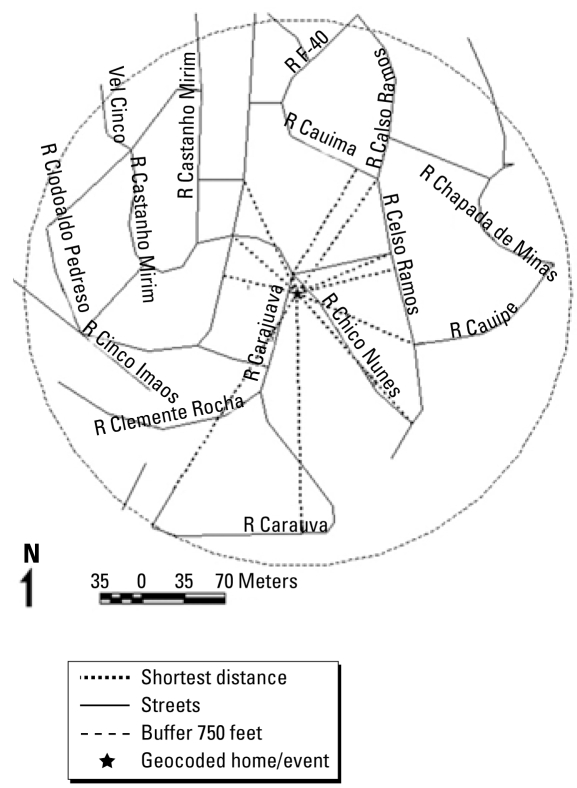
Representation of the calculation of shortest distances between location of a case or control and all roadways in a radius of 750 feet.

Using a method similar to that of Pearson et al. (2000), we calculated the DWTD for each subject, which we used as an indicator of exposure to traffic-related air pollutants close to their homes. More specifically, we delineated a radius of 750 feet (228.6 m) around the home of each case and control and identified all streets contained in this buffer for later calculation of the perpendicular distance between each street and home. We applied a model also developed by Pearson et al. (2000) to estimate the dispersion of pollutants emitted by cars inside this circumference. This model assumes that 96% of the total pollutants emitted disperse up to 500 feet (152.4 m) from the road according to the equation


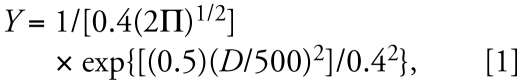


where *D* is the shortest distance from the home to each street within the delimited area (Figure 1) and *Y* is the value used to weight the traffic flow for each street. We then summed the weighted values of the traffic flow for all streets surrounding the subject’s home.

### Analysis

We fit separate models for fetal and neonatal deaths to explore in detail the effect of our indicator of exposure to traffic pollution (DWTD) on each outcome. We grouped the DWTD values into quartiles derived from its distribution for all subjects and examined their association with each outcome using logistic regression analysis.

Based on previous analyses of these data (Almeida et al. 2007; Schoeps et al. 2007), we adjusted our models for covariates representing four different groups of potential risk factors for perinatal mortality according to a hierarchical conceptual framework. This included *a*) socioeconomic and demographic characteristics of mothers and families (family income, head of the household education, maternal occupation, maternal marital status and length of union, housing type, and crowding), *b*) characteristics of mothers before pregnancy (age, parity, previous low-birth-weight infant), *c*) conditions during pregnancy (vaginal bleeding, hypertension, diabetes, cigarette and alcohol consumption, planning of the pregnancy, and prenatal care), and *d*) characteristics of delivery and the fetus (sex, birth weight, gestational age, IUGR, presence of fetal malformation, and problems during delivery).

We combined marital status and length of union into one variable. We grouped maternal occupation during the pregnancy in a dichotomous variable “working” and “not working” (unemployed, retired, housewife, student, or without activity). Housing type refers to the construction material (brickwork or other materials, e.g., wood, usually found in more precarious housing). We based problems during delivery on maternal report (yes/no), and they refer mostly to hemorrhage and eclampsia.

We used univariate regression to obtain crude odds ratios (OR) and to select the most important covariates (*p* < 0.20) from each of the four groups to be included in the multivariate logistic models. We kept variables in the final models if they attained statistical significance (*p* < 0.05) or if we considered them important potential confounders of the association between our indicator of exposure and perinatal mortality, regardless of their significance. We did not include birth weight, gestational age, and IUGR in our models because these conditions may lie in the causal pathway between traffic-related air pollution and perinatal mortality, so it would not be adequate to control for them.

## Results

Of the total 749 eligible events (378 cases and 371 controls), we omitted 60 cases (15.9%) and 58 controls (15.6%) because of incorrect residential address (16 cases and 8 controls), mother refusal (12 cases and 9 controls), event without hospital record (21 cases and 19 controls), or hospital refused access to records (11 cases and 22 controls).

Therefore, we obtained a sample of 631 events in the period between August 2000 and January 2001, corresponding to 318 perinatal deaths (146 early neonatal and 172 fetal deaths) and 313 unpaired controls. Of the total 172 fetal deaths, we excluded 10 from the study because eight were intrapartum deaths (based on obstetric records), one event did not discriminate sex, and another could not be geocoded, leaving 162 fetal deaths for analysis.

The distribution of the DWTD for this population was highly skewed, with a minimum of 6 vehicles/hr and maximum of > 10,000 vehicles/hr (median of 45.3 vehicles/hr) (Table 1). On average, cases had higher values than did controls for this indicator, although the difference was not statistically significant. In addition, DWTD values were higher among early neonatal deaths than among fetal deaths.

Table 2 summarizes the demographic characteristics of cases and controls as well as maternal conditions during pregnancy. As expected, proportions of low birth weight and prematurity were much higher among cases than among controls. Mothers of early neonatal deaths were younger than other groups, lived in more crowded environments, and had worse social conditions than did mothers of controls. There were also a greater proportion of single mothers among cases, especially for fetal deaths. In addition, mothers of cases had higher parity and greater proportions of unplanned pregnancies and previous low-birth-weight children. They also had worse antenatal care, smoked substantially more, and exhibited higher rates of morbidities during pregnancy than did mothers of controls (Table 2).

As expected, low birth weight and prematurity exhibited the strongest association with perinatal mortality, followed by morbidities developed during pregnancy such as diabetes, vaginal bleeding (possible proxy for placenta previa), and hypertension. The presence of congenital anomalies yielded a much higher risk for neonatal mortality (OR = 13.6) than for fetal mortality (OR = 3.2).

The univariate analysis of the association between DWTD and perinatal mortality showed that the OR for neonatal deaths increased with DWTD quartiles, and in the highest quartile we observed a 78% increase in risk for this outcome [OR = 1.78; 95% confidence interval (CI), 1.01–3.16]. For fetal deaths we did not observe such a consistent increase in risk (OR in the highest quartile = 1.35; 95% CI, 0.79–2.30).

The multivariate analysis showed that length of union, previous low-birth-weight infant, vaginal bleeding, hypertension, antenatal care, problems during delivery, and congenital anomalies were all associated with fetal deaths. The OR for fetal deaths associated with the traffic-related air pollution indicator decreased after adjustment. The adjusted OR for the highest category of DWTD was 1.20 (95% CI, 0.65–2.24) (Table 3).

For neonatal deaths, length of union, housing type, maternal age, previous low-birth-weight infant, vaginal bleeding, antenatal care, sex, congenital anomalies, and presence of problems during delivery remained in the multivariate analysis as significant risk factors. The association of these deaths with DWTD adjusted for covariates exhibited a pattern similar to that in the univariate analysis—that is, an increase in risk of neonatal deaths for mothers in the higher exposure categories. However, the trend observed in the univariate analysis was no longer clear. The adjusted OR in the higher category of exposure was 1.47 (95% CI, 0.67–3.19) (Table 4).

## Discussion

To our knowledge, this is the first study to evaluate the association between perinatal mortality and exposure of pregnant women and newborns to pollutants from heavy-traffic roadways in the vicinity of their homes. We divided perinatal deaths into two components, fetal and early neonatal deaths, given that marked differences in the etiology of their determinants have been observed (Kramer et al. 2002).

We observed an association of fetal and early neonatal deaths with our indicator of traffic-related air pollution. Mothers in the highest categories of exposure exhibited increased risk, although this association was stronger for neonatal than for fetal deaths because risks were higher for neonatal deaths in all categories of exposure. In addition, adjusted analysis for fetal deaths exhibited an increase in risk only for the higher category of exposure to traffic-related air pollution.

For early neonatal deaths, results suggest that women with a higher load of exposure to traffic have a nearly 50% increase in risk compared with less exposed mothers. We also observed an increasing trend in the univariate analysis, but it disappeared after adjustment for other covariates.

The case–control study that provided the data for our analysis was not originally planned to evaluate the role in perinatal mortality of living close to heavy-traffic roadways, and thus potentially being exposed to higher levels of motor vehicle exhaust. Therefore, the sample size obtained may not have provided sufficient power to explore this relationship.

Although birth weight, gestational age, and IUGR are important risk factors for perinatal mortality, we did not include them in our final models. Studies have shown that these conditions are also associated with exposure to air pollution (Ritz and Yu 1999; Ritz et al. 2000), so it is very likely that they lie in the causal pathway between traffic-related air pollution and perinatal deaths. Other pregnancy complications such as hypertension and diabetes may also make the fetus more susceptible to the additional insult of air pollution, but it is less likely that they are in the causal pathway because, in our study, these are conditions developed during pregnancy and thus essentially are associated with the pregnancy itself. In all these cases, these variables might modify the effect of exposure. However, we did not have sufficient statistical power to evaluate this hypothesis.

Another limitation of this study is the potential for misclassification of the exposure of pregnant women to traffic-related air pollution. Because we estimated this exposure based on the home address, it is possible that we incorrectly classified those who spent most of their time during pregnancy in another location (e.g., work). It is also possible that some of these women might have moved during pregnancy. We do not have data on mobility, but according to the information on maternal occupation during pregnancy, 58% of mothers of controls, 63% of mothers of neonatal deaths, and 51% of mothers of fetal deaths were housewives, unemployed, retired, or students, which means they might have stayed at home most of the time during pregnancy. Even for those who worked, it is likely that they might have stayed mostly at home during the final months of pregnancy, thus enhancing their exposure to the local traffic-related air pollutants. It should also be noted that São Paulo has a mild climate, and people keep windows open throughout the year. Therefore, a significant portion of outdoor pollution from traffic exhaust penetrates indoors.

In addition, we estimated the DWTD measures without knowledge of the case–control status, so any errors in the DWTD measurement are likely to be nondifferential, resulting in an underestimation of the risk associated with traffic-related air pollution.

Other sources of imprecision in our estimate of exposure include the lack of information on wind patterns and local topography, which may alter the spatial distribution of air pollutants (e.g., traffic-related air pollution may be different for those living upwind or downwind) and the different types of vehicles passing in a given street, such as gasoline-, ethanol-, or diesel-fueled vehicles, which have different emission profiles. These are all potential sources of variability in our estimates of exposure, but again, one does not expect them to be differentially distributed between cases and controls.

Despite these limitations, this indicator of exposure to traffic-related pollutants has been applied in different studies (Bayer-Oglesby et al. 2006; Ciccone et al. 1998; Kramer et al. 2000; Pearson et al. 2000; Wilhelm and Ritz 2003) because it is relatively easy to interpret and provides an account of the importance of living close to busy roads, and therefore exposure to vehicular emissions. Other studies have shown that concentrations of pollutants near roadways are well correlated with traffic counts (Kinney et al. 2000; Lena et al. 2002), so the DWTD model can be used as an indicator of population exposure to urban air pollutants.

The original study was carefully designed to avoid selection bias, because all cases of perinatal deaths during the study period were included and controls were randomly selected among survivors of the neonatal period identified from live-born birth certificates. We compared the data on sex, birth weight, and gestational age available in the birth certificates for those whom we did not include in the study (~16% of cases and controls) with the included infants and found no statistical significant differences among them, confirming that there was no selection bias.

We used extensive collected data in this study, including variables related to socioeconomic conditions, maternal reproductive history and risk factors, and outcome of previous and present pregnancy and delivery. We assessed important risk factors for perinatal deaths and identified and controlled for potential confounding variables. Nevertheless, we cannot rule out some residual confounding by other factors for which we did not have data, such as meteorologic aspects. Background air pollution is correlated with temperature and/ or other meteorologic parameters, and its effect on health might vary according to season. However, we could not examine this in detail because we selected the events of this study from a period of only 6 months.

Few other studies have investigated the association between deaths in the perinatal period and air pollution (Hajat et al. 2007; Lin et al. 2004; Nishioka et al. 2000; Pereira et al. 1998). Most studies have used a time-series approach and therefore examined the effect of air pollution on day(s) before the event (temporal scale). In addition, they relied on air quality monitoring sites for assessment of exposure to urban air pollution. In general, they all found statistically significant associations between daily counts of deaths (late fetal, neonatal, infant) and the average concentration of pollutants in the atmosphere. Pereira et al. (1998), in a study conducted in São Paulo, also showed that carboxyhemoglobin levels of blood collected from the umbilical cord of nonsmoking mothers were correlated with environmental carbon monoxide. These studies, although not directly comparable with the present study, which we based on spatial comparisons, suggest that fetuses and newborns may suffer from the consequences of a contaminated environment.

Potential biologic mechanisms behind this association may involve an effect of air pollution on the placenta, embryo, maternal immunologic system, ovarian–hypothalamic axis, and the induction of IUGR, which can lead to a more vulnerable fetus. It is possible that certain toxics emitted in motor vehicle exhaust, such as polycyclic aromatic hydrocarbons (PAHs), may be responsible for these adverse birth outcomes. Exposure to PAHs during pregnancy has been demonstrated to alter levels of serum progesterone and estrogens, decrease survival of rats, and relate to endocrine disorders in rodents (Takeda et al. 2004). Air particles may be associated with changes in plasma viscosity (Peters et al. 1997), inflammatory process (Dubowsky et al. 2006), and elevation in blood pressure in susceptible populations (Brook 2005). Carbon monoxide easily crosses the placental barrier, coming into contact with the fetus, leading to a rapid accumulation of carboxyhemoglobin, with subsequent reduction in oxygen transportation by blood (Aleixo Neto 1990).

The results of the present study suggest that the early neonatal component of perinatal mortality may be associated with mothers’ exposure to air pollution from traffic near their homes. Although we could not provide strong evidence of such association, the consistent literature and the biologic plausibility indicate that motor vehicle exhaust exposures may be important for this outcome.

## Figures and Tables

**Table 1:** Descriptive statistics of the DWTD values according to case and control categories.

Category	Mean ± SD	Median	25th percentile	75th percentile
All events	504 ± 1225.8	45.3	6.1	370.2
Cases	565.3 ± 1361.8	52.6	7.4	434.1
Fetal	530.9 ± 1364.3	45.1	6.8	426.5
Early neonatal	626.8 ± 1398.6	72.5	8.5	586.6
Controls	441.9 ± 1069.3	35.8	5.4	340.9

**Table 2:** Subjects [no. (%)] and univariate ORs according to demographic and socioeconomic characteristics, maternal conditions before and during pregnancy, and characteristics of delivery and fetus.

		Fetal deaths		Neonatal deaths	
Variable	Controls [no. (%)]	no. (%)	OR	no. (%)	OR
Socioeconomic and demographic characteristics					
Per capita family income at Brazilian minimum wage					
> 10	38 (12.6)	14 (9.1)	1	13 (9.4)	1
3–9	143 (47.5)	74 (48.4)	1.4	61 (44.2)	1.25
≤2	120 (39.9)	65 (42.5)	1.47	64 (46.4)	1.56
			*p* = 0.353		*p* = 0.157
Head-of-family education (years)					
< 12	299 (95.5)	159 (98.1)	1	144 (98.6)	3.37
≥12	14 (4.5)	3 (1.9)	2.48	2 (1.4)	1
			*p* = 0.158		*p* = 0.111
Maternal occupation					
Working	121 (39.7)	76 (51.7)	1	61 (46.2)	1
Not working	184 (60.3)	71 (48.3)	0.66	71 (53.8)	0.76
			*p* = 0.042		*p* = 0.203
Marital status and length of union (years)					
≥1	243 (77.6)	94 (58.0)	1	96 (65.8)	1
< 1	22 (7.0)	20 (12.4)	2.35	17 (11.6)	1.96
Single mother	48 (15.4)	48 (29.6)	2.58	33 (22.6)	1.74
			*p* < 0.001		*p* = 0.014
Housing type					
Brickwork	245 (78.3)	133 (82.0)	1	93 (63.7)	1
Other materials	68 (21.7)	29 (18.0)	0.78	53 (36.3)	2.05
			*p* = 0.328		*p* = 0.001
No. occupants per room					
< 1	92 (29.3)	42 (25.9)	1	31 (21.2)	1
1–2	203 (64.9)	104 (64.2)	1.12	94 (64.4)	1.37
3–4	18 (5.8)	16 (9.9)	1.92	21 (14.4)	3.46
			*p* = 0.159		*p* = 0.003
Maternal characteristics before pregnancy					
Age (years)					
> 20	261 (83.4)	132 (81.5)	1	102 (69.9)	1
≤20	52 (16.6)	30 (18.5)	1.14	44 (30.1)	2.16
			*p* = 0.603		*p* = 0.001
Parity					
2nd or 3rd	155 (49.5)	79 (48.8)	1	53 (36.3)	1
1st	128 (40.9)	61 (37.6)	0.93	69 (47.3)	0.93
≥4th	30 (9.6)	22 (13.6)	1.44	24 (16.4)	1.8
			*p* = 0.467		*p* = 0.185
Previous low-birth-weight infant					
No	281 (89.8)	133 (82.1)	1	111 (76.0)	1
Yes	32 (10.2)	29 (17.9)	1.91	35 (24.0)	2.77
			*p* = 0.019		*p* < 0.001
Conditions during pregnancy					
Vaginal bleeding					
No	307 (98.1)	140 (86.4)	1	119 (81.5)	1
Yes	6 (1.9)	22 (13.6)	8.04	27 (18.5)	11.61
			*p* < 0.001		*p* < 0.001
Hypertension					
No	291 (93.0)	109 (67.3)	1	124 (84.9)	1
Yes	22 (7.0)	53 (32.7)	6.43	22 (15.1)	2.35
			*p* < 0.001		*p* = 0.008
Conditions during pregnancy					
Diabetes					
No	309 (99.7)	150 (95.5)	1	140 (97.9)	1
Yes	1 (0.3)	7 (4.5)	14.42	3 (2.1)	6.62
			*p* = 0.013		*p* = 0.103
Maternal smoking					
No	254 (81.2)	118 (72.8)	1	98 (67.1)	1
Yes	59 (18.8)	44 (27.2)	1.6	48 (32.9)	2.11
			*p* = 0.038		*p* = 0.001
Maternal alcohol consumption					
No	257 (82.1)	172 (75.3)	1	120 (82.2)	1
Yes	56 (17.9)	40 (24.7)	1.5	26 (17.8)	0.99
			*p* = 0.081		*p* = 0.983
Planned pregnancy					
Yes	116 (37.1)	46 (28.4)	1	39 (26.7)	1
No	197 (62.9)	116 (71.6)	1.48	107 (73.3)	1.61
			*p* = 0.06		*p* = 0.03
Antenatal care					
Adequate	232 (74.1)	83 (51.2)	1	73 (50.0)	1
Inadequate	77 (24.6)	70 (43.2)	2.54	52 (35.6)	2.15
None	4 (1.3)	9 (5.6)	6.29	21 (14.4)	16.68
			*p* < 0.001		*p* < 0.001
Characteristics of delivery and the fetus					
Sex					
Female	156 (49.8)	72 (44.4)	1	58 (39.7)	1
Male	157 (50.2)	90 (55.6)	1.14	88 (60.3)	1.51
			*p* = 0.265		*p* = 0.044
Birth weight (g)					
≥2,500	288 (92.0)	38 (24.5)	1	27 (18.5)	1
< 2,500	25 (8.0)	117 (75.5)	37.6	119 (81.5)	50.77
			*p* < 0.001		*p* < 0.001
Length of gestation (weeks)					
≥37	269 (85.9)	51 (32.7)	1	28 (19.2)	1
< 37	44 (14.1)	105 (67.3)	12.59	118 (80.8)	25.76
			*p* < 0.001		*p* < 0.001
Congenital anomaly					
No	304 (97.1)	148 (91.4)	1	104 (71.2)	1
Yes	9 (2.9)	14 (8.6)	3.19	42 (28.8)	13.64
			*p* = 0.008		*p* < 0.001
Problems during delivery					
No	289 (92.3)	123 (75.9)	1	114 (78.6)	1
Yes	24 (7.7)	39 (24.1)	3.82	31 (21.4)	3.27
			*p* < 0.001		*p* < 0.001
DWTD					
≤6.0	89 (28.4)	38 (23.5)	1	28 (19.1)	1
> 6.0 to ≤45.3	75 (24.0)	43 (26.5)	1.34	38 (26.1)	1.61
> 45.3 to ≤370.2	76 (24.3)	39 (24.1)	1.2	39 (26.7)	1.63
> 370.2	73 (23.3)	42 (25.9)	1.35	41 (28.1)	1.78
			*p* = 0.36		*p* = 0.06

**Table 3:** Adjusted OR (95% CI) for fetal deaths for each covariate included in the adjusted model.

Covariate	Adjusted OR (95% CI)	*p*-Value
Marital status and length of union (years)		
≥1	1.00	0.001
< 1	2.80 (1.32–5.96)	
Single mother	2.24 (1.30–3.85)	
Previous low-birth-weight infant		
No	1.00	0.021
Yes	2.19 (1.14–4.19)	
Conditions during pregnancy		
Vaginal bleeding		
No	1.00	0.001
Yes	6.14 (2.11–17.85)	
Hypertension		
No	1.00	< 0.001
Yes	6.61 (3.59–12.16)	
Antenatal care		
Adequate	1.00	< 0.001
Inadequate	2.33 (1.46–3.73)	
None	3.57 (0.83–15.37)	
Problems during delivery		
No	1.00	< 0.001
Yes	3.31 (1.75–6.29)	
Congenital malformation		
No	1.00	0.004
Yes	4.17 (1.58–11.05)	
DWTD		
≤6.0	1.00	0.709
> 6.0 and ≤45.3	1.06 (0.57–1.96)	
> 45.3 and ≤370.2	0.92 (0.48–1.77)	
> 370.2 and ≤10,810.9	1.20 (0.65–2.24)	

**Table 4:** Adjusted OR (95% CI) for neonatal deaths for each covariate included in the adjusted model.

Covariate	Adjusted OR (95% CI)	*p*-Value
Length of union (years)		
≥1	1.00	0.024
< 1	3.94 (1.61–9.65)	
Single mother	1.89 (0.95–3.76)	
Housing type		
Brickwork	1.00	0.049
Other materials	1.64 (0.93–2.91)	
Maternal age		
> 20	1.00	0.022
≤ 20	1.88 (1.01–3.51)	
Previous low-birth-weight infant		
No	1.00	< 0.001
Yes	4.82 (2.44–9.53)	
Vaginal bleeding		
No	1.00	< 0.001
Yes	12.71 (4.28–37.68)	
Antenatal care		
Adequate	1.00	< 0.001
Inadequate	2.12 (1.20–3.75)	
None	36.09 (10.30–126.48)	
Sex		
Female	1	0.023
Male	1.91 (1.12–3.27)	
Congenital malformation		
No	1.00	< 0.001
Yes	27.85 (11.34–68.41)	
Problems during delivery		
No	1.00	< 0.001
Yes	5.17 (2.41–11.07)	
DWTD		
≤6.0	1.00	0.215
> 6.0 and ≤45.3	1.46 (0.67–3.18)	
> 45.3 and ≤370.2	2.82 (1.32–6.03)	
> 370.2 and ≤10810.9	1.47 (0.67–3.19)	
